# Treatment of Recurrent Eczema Herpeticum in Pregnancy With Acyclovir

**DOI:** 10.1155/S1064744996000452

**Published:** 1996

**Authors:** Richard A. Latta, David A. Baker

**Affiliations:** 1 Division of Maternal-Fetal Medicine Abington Memorial Hospital Abington PA 19001 USA; 2 Division of Maternal-Fetal Medicine State University of New York at Stony Brook Stony Brook NY USA

## Abstract

*Background:* Eczema herpeticum is an uncommon manifestation of an infection with herpes simplex virus (HSV). The disease is primarily seen in patients with histories of atopic eczema. Eczema herpeticum may be a life-threatening illness, but the mortality is felt to be <10% with modern antiviral and antibacterial agents. The use of acyclovir for other viral infections secondary to herpesvirus in pregnancy has been well documented. The authors now present a case report of eczema herpeticum treated with acyclovir during pregnancy.

*Case:* A patient with a history of eczema herpeticum presented in pregnancy with a recurrence. She was successfully treated with intravenous (IV) acyclovir with good maternal and fetal outcome.

*Conclusion:* Acyclovir may be utilized in pregnancy for several manifestations of HSV including eczema herpeticum.


Infectious Diseases in Obstetrics and Gynecology 4:239–242 (1996)
© 1997 Wiley-Liss, Inc.

### Treatment of Recurrent Eczema Herpeticum in Pregnancy With Acyclovir

**Richard A. Latta and David A. Baker**

*Division of Maternal-Fetal Medicine, Abington Memorial Hospital, Abington, PA (R.A.L.); Division of Maternal-Fetal Medicine, State University of New York at Stony Brook, Stony Brook, NY (D.A.B.)*

ABSTRACT

*Background:* Eczema herpeticum is an uncommon manifestation of an infection with herpes simplex virus (HSV). The disease is primarily seen in patients with histories of atopic eczema. Eczema herpeticum may be a life-threatening illness, but the mortality is felt to be <10% with modern antiviral and antibacterial agents. The use of acyclovir for other viral infections secondary to herpesvirus in pregnancy has been well documented. The authors now present a case report of eczema herpeticum treated with acyclovir during pregnancy.

*Case:* A patient with a history of eczema herpeticum presented in pregnancy with a recurrence. She was successfully treated with intravenous (IV) acyclovir with good maternal and fetal outcome.

*Conclusion:* Acyclovir may be utilized in pregnancy for several manifestations of HSV including eczema herpeticum. © 1997 Wiley-Liss, Inc.

KEY WORDS

Herpes simplex virus, Kaposi's varicelliform eruption, rash, dermatitis

Eczema herpeticum, also known as Kaposi's varicelliform eruption, is a viral vesiculopustular exanthem secondary to an infection with herpes simplex virus (HSV). HSV type I is primarily the responsible agent, although type II has also been described.[1] The disproportionate number of HSV infections ascribed to type I may be secondary to an underestimation because typing of the virus is usually not performed.[2] Patients with atopic eczema appear to be particularly susceptible.[3] Patients with other dermatologic conditions including autosomal dominant ichthyosis vulgaris, Darier's disease, familial benign pemphigus, pemphigus foliaceous, and congenital ichthyosiform erythroderma are predisposed to the disorder.

The manifestations of eczema herpeticum may range from a mild transient rash to a fatal illness. The cause of fatalities seems to be an extensive viremia with involvement of the internal organs by a secondary bacterial infection, usually β-hemolytic streptococcus or *Staphylococcus aureus*.[2]

### Case Report

B. A., a 20-year-old white female, presented to the emergency room with a recurrent facial rash. Her medical history was significant for asthma requiring several hospital admissions per year. She had had eczema herpeticum diagnosed 1 year earlier. Her prior episode began when she noted painful, raised flesh-colored blisters on her lips. The legions spread across her cheeks, nose, and forehead. A skin culture was positive for HSV. An eye exudate culture revealed *S. aureus*. She was treated with intravenous (IV) nafcillin and acyclovir and discharged home with the rash almost completely resolved. An ophthalmologic examination in the hospital revealed

Address correspondence/reprint requests to Dr. Richard A. Latta, Suite 119, Levy Medical Plaza, Abington Memorial Hospital, Abington, PA 19001.

Received April 15, 1996
Accepted July 8, 1996

ocular HSV involvement. The ocular involvement resolved without residual impairment. She was discharged home on oral dicloxacillin and acyclovir. She presented to the emergency room 1 year later with a recurrent facial rash. She had had a positive pregnancy test approximately 6 weeks prior to her presentation, although she was uncertain of her last menstrual period.

Her facial rash began approximately 36 h prior to her presentation. She had diffuse, mildly tender, bilateral anterior cervical lymphadenopathy. Her skin demonstrated a raised erythematous rash coalescing over the face with angioedema. There was no eye involvement or extension beyond the face on admission. The lesions were red and raised with crusting (Fig. 1). They were of the same initial distribution and character as her previous rash from eczema herpeticum. She was afebrile. A diffuse maculopapular rash with tender crusting lesions was noted. Aerobic, anaerobic, and HSV cultures were obtained. A sonogram revealed a 12-week single viable intrauterine pregnancy. The patient was started on IV acyclovir with rapid resolution of the lesions. The HSV culture returned positive. She was discharged home on oral acyclovir (200 mg 5 times a day for 7 days) after 5 days of hospitalization and marked improvement in the rash.

The remainder of her pregnancy was complicated by an admission for bronchitis and asthma at 37 weeks gestation. Her fundal height grew appropriately throughout her pregnancy. She had normal sonograms at 28 and 37 weeks gestation. She presented to labor and delivery at 38-2/7 weeks gestation with rupture of the membranes. She spontaneously began to labor. After pitocin augmentation, she delivered a viable female infant with a birth weight of 3,380 g and Apgars of 9/9. She and her infant had uncomplicated postpartum courses. They are currently doing well.

### DISCUSSION

We describe a case of eczema herpeticum during pregnancy. Eczema herpeticum is a disease most frequently caused by HSV type I but sometimes HSV type II. Swart and colleagues[4] previously described the treatment of 3 cases of eczema herpeticum with acyclovir. There are only 2 other reports of acyclovir treatment of eczema herpeticum during pregnancy.[5,6] Eczema herpeticum is a disease with widely ranging degrees of severity, from very mild symptoms to a rapidly fatal illness. Since the utilization of acyclovir for other herpes-related illnesses in pregnancy has been successful and the pharmacokinetics have been previously delineated, we used IV acyclovir with good therapeutic response in a patient with eczema herpeticum. This pregnant patient subsequently delivered at term with an uncomplicated postpartum course for mother and child.

Acyclovir is now well established in the armamentarium used against HSV infections. It has been shown to be effective in treating primary, recrudescent, and life-threatening HSV infections in normal and immunocompromised adults, as well as newborn infants. There are numerous case reports of the use of acyclovir in pregnancy. The pharmacokinetics of acyclovir in pregnancy have also been examined.[7] Brown and associates[8] presented information on the oral administration of acyclovir to women with histories of recurrent genital herpes at the Interscience Conference on Antimicrobial Agents and Chemotherapy in New York in 1987. The peak levels observed in term pregnant patients were somewhat lower than those observed in nonpregnant patients after oral dosing with 200 mg of acyclovir. The ratio of plasma concentration of acyclovir in mothers-to-newborns was 1.12. Lau and colleagues[9] studied the plasma concentrations of acyclovir in breast-feeding women. They calculated a plasma-to-milk ratio of 0.15. This finding implies that the concentrations of breast milk exceeded those of the maternal plasma, therefore, appearing to be concentrated in breast milk.

Toxicities and teratogenesis have been studied in animal models.[10,11] Chromosomal damage was observed in cultured human lymphocytes at 200 μg/ml, which is 25 times the peak levels achieved with IV dosing in pregnancy but not ≤125 μg/ml. An ongoing registry established by the Burroughs Wellcome (now Glaxo Wellcome) Company continues to accumulate the results of pregnancies prospectively exposed to acyclovir. The most recent registry interim report listed 847 prospectively exposed pregnancies (personal communication). The information derived from this registry will be useful in the evaluation of any clinically significant effects of acyclovir in pregnancy.

Acyclovir has been used in primary genital herpes during pregnancy. Several studies have indicated that primary genital herpes is associated with

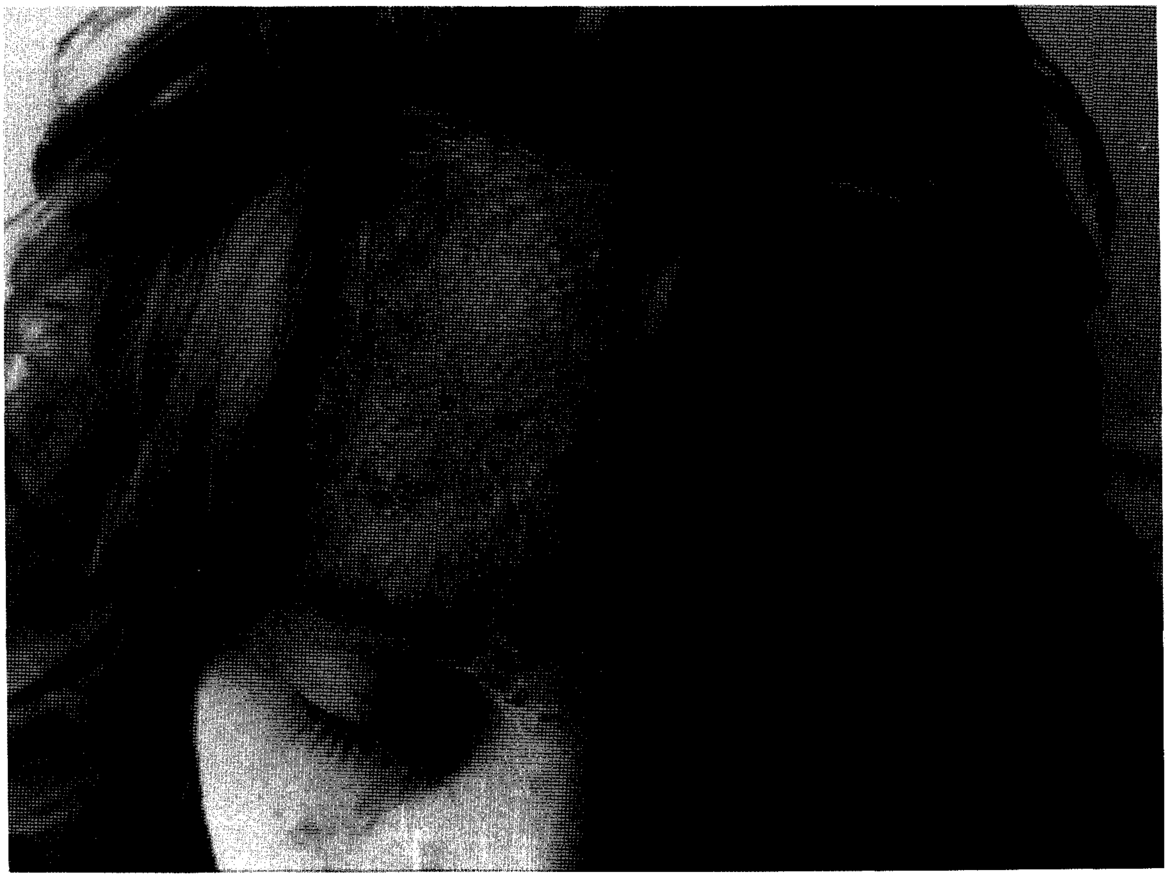

Fig. 1. Lesions of eczema herpeticum. Note the extensive involvement and epithelial destruction caused by HSV.

a higher incidence of obstetric and perinatal complications. The predominant mechanism of poor outcome appears to be an increased incidence of premature labor and delivery.[12] Case reports have also described the use of acyclovir in primary genital herpes in patients with premature rupture of the membranes.[13]

Life-threatening, disseminated HSV infection in pregnancy has been treated with acyclovir.[14] This form of HSV infection usually occurs in the immunosuppressed or immunocompromised host, but can occur in an immunocompetent adult. It seems likely in the current setting of an HIV epidemic that this problem may be faced in ever-increasing numbers of pregnant individuals. Other potentially life-threatening maternal diseases including acute varicella pneumonia complicating pregnancy have also been treated with IV acyclovir.[15]

Acyclovir is an effective antiviral agent in the treatment of serious herpes infections. There appears to be a low morbidity related to its use during pregnancy. The utilization of acyclovir for serious disease with potentially life-threatening consequences during pregnancy appears indicated. We believe that the treatment of eczema herpeticum is consistent with the CDC's 1989 treatment guidelines which states that, "In the presence of life-threatening maternal HSV infections, acyclovir administered IV is probably of value."[16]

### REFERENCES

1. Hazen PG, Bennet-Epprs R: Eczema herpeticum caused by herpesvirus type 2. Arch Dermatol 113:1085–1086, 1977.
2. David TJ, Longson M: Herpes simplex infections in atopic eczema. Arch Dis Child 60:338–343, 1985.
3. Zachariae H: Herpesvirus infection in man. Scand J Infect Dis Suppl 47:44–50, 1985.
4. Swart RNJ, Vermeer BJ, van Der Meer JWM, Enschede FAJ, Versteeg J: Treatment of eczema herpeticum with acyclovir. Arch Dermatol 119:13–16, 1983.

5. Garland SM, Hill PJ: Eczema herpeticum in pregnancy successfully treated with acyclovir. Aust NZ J Obstet Gynaecol 34:214–215, 1994.
6. Wollenberg A, Degitz K: Herpetic eczema in pregnancy. Dtsch Med Wochenschr 120:1395–1398, 1995.
7. Schaeffer HJ: Acyclovir chemistry and spectrum of activity. Am J Med 73:3–4, 1982.
8. Brown ZA, Corey L, Unadkat J, Schumann L, Arvin A, Henleigh P, Prober C, Bryson Y, Connor J: Pharmacokinetics of ACV in the term human pregnancy and neonate. Abstract, 27th Interscience Conference on Antimicrobial Agent and Chemotherapy, New York, October 4–7, 1987.
9. Lau RJ, Emergy MG, Galinsky RE: Unexpected accumulation of acyclovir in breast milk with estimation of infant exposure. Obstet Gynecol 69:468–470, 1987.
10. Moore HL, Szczech GM, Rodwell DE, Kapp RW, DeMiranda P, Tucker WE: Preclinical toxicology studies with acyclovir: Teratologic, reproductive, and neonatal tests. Fundam Appl Toxicol 3:560–568, 1983.
11. Clive D, Turner NT, Hozier J, Baston AG, Tucker WE: Preclinical toxicology studies with acyclovir: Genetic toxicity test. Fundam Appl Toxicol 3:587–602, 1983.
12. Nahmia AJ, Josey WE, Naib ZM, Freeman MG, Fernandez RG, Wheeler JH. Perinatal risk associated with maternal genital herpes simplex virus infection. Am J Obstet Gynecol 110:825–837, 1971.
13. Utley K, Bromberger P, Wagner L, Schneider H: Management of primary herpes in pregnancy complicated by ruptured membranes and extreme prematurity: Case Report. Am J Obstet Gynecol 69:471–473, 1987.
14. Hankley GJ, Bucens MR, Chambers JSW: Herpes simplex encephalitis in the third trimester of pregnancy: Successful outcome for mother and child. Neurology 37:1534–1537, 18987.
15. Hankins GDV, Gilstrap LC, Patterson AR: Acyclovir treatment of varicella pneumonia in pregnancy. Crit Care Med 15:336–337, 1987.
16. U.S. Department of Health and Human Services, Public Health Service, Center for Disease Control: Sexually Transmitted Treatment Guidelines, 1989.
